# Using monoclonal antibodies to label living root hairs: a novel tool for studying cell wall microarchitecture and dynamics in *Arabidopsis*

**DOI:** 10.1186/1746-4811-10-30

**Published:** 2014-10-02

**Authors:** Emily R Larson, Mary L Tierney, Berke Tinaz, David S Domozych

**Affiliations:** Cellular, Molecular, and Biomedical Science Program, University of Vermont, Burlington, VT USA; Department of Plant Biology, University of Vermont, Burlington, VT USA; Department of Biology, Skidmore College, Saratoga Springs, NY USA; Laboratory of Plant Physiology and Biophysics, Institute of Molecular Cell and Systems Biology, University of Glasgow, Bower Building, Glasgow, G12 8QQ UK

**Keywords:** Arabidopsis, Root hairs, Live cell labeling, Immunocytochemistry, Polar growth, Cell wall

## Abstract

**Background:**

The *Arabidopsis* root hair represents a valuable cell model for elucidating polar expansion mechanisms in plant cells and the overall biology of roots. The deposition and development of the cell wall is central to the root hair expansion apparatus. During this process, incorporation of specific wall polymers into the growing wall architecture constitutes a critical spatio-temporal event that controls hair size and growth rate and one that is closely coordinated with the cell’s endomembrane, cytoskeletal and signal transduction apparatuses.

**Results:**

In this study, the protocol for live cell labeling of roots with monoclonal antibodies that bind to specific wall polymers is presented. This method allows for rapid assessment of root hair cell wall composition during development and assists in describing changes to cell wall composition in transgenic mutant lines. Enzymatic “unmasking” of specific polymers prior to labeling allows for refined interpretation of cell wall chemistry. Live cell immunofluorescence data may also be correlated with transmission electron microscopy-based immunogold labeling.

**Conclusions:**

Live *Arabidopsis* root hairs may be labeled with cell wall polymer-specific antibodies. This methodology allows for direct visualization of cell wall dynamics throughout development in stable transgenic plant lines. It also provides an important new tool in the elucidation of the specific interactions occurring between membrane trafficking networks, cytoskeleton and the cell wall deposition/remodeling mechanism.

## Background

The expansion dynamics of a plant cell are directly controlled by the microarchitecture of its cell wall. Modulations to cell wall constituents via new polymer deposition and/or remodeling of pre-existing polymers create loosened or “softened” zones with less tensile strength throughout the wall or at specific sites therein. Internal turgor pressure generates the non-vectorial force against the wall that drives the expansion process at these softened zones [1, 2]. Many plant cells exhibit *diffusive* growth whereby growth is roughly equivalent on all faces of the expanding cell. However, other cell types grow in a *polar* fashion where wall and cell expansion are focused at a specific point or front [3, 4]. Tensile resistance of the wall to turgor is less at this front, that in turn, allows for a localized but controlled cell expansion. At other regions of the cell the wall retains sufficient tensile strength to resist turgor-driven pressure. This type of growth often leads to distinct tubular shapes, as exemplified by pollen tubes, root hairs and moss protonemata.

The most well studied polar expansion system in land plants is the pollen tube [5, 6]. At the tip of a growing tube, pectin dynamics create a less-rigid wall that promotes polarized expansion driven by turgor [7, 8]. In the shank areas adjacent to the tip, modulation of wall chemistry creates a rigid matrix organization that is resistant to turgor pressure. This includes de-esterification of the pectin followed by calcium (Ca^2+^) complexing to yield a rigid gel and the addition of ß(1–3)-glucan (callose) and ß(1–4)-glucan (cellulose) to the wall.

Root hairs represent specialized extensions of the root epidermis that are also formed by polar expansion. These structures are profoundly important for the survival of a plant as they are critical for the uptake of water and minerals and are involved in the establishment of symbiotic associations with resident microbiota of soils [9–11]. Surprisingly, far less is known about wall composition and architecture, and its role in polar expansion of root hairs than for other polar expanding plant cells. This is due to the exceptionally fragile nature of the hairs, especially when handling during experimental manipulation, and the difficulty in obtaining sufficient amounts of root hair wall material for biochemical studies. Presently, it is thought that cellulose microfibril arrangement is random at the growing root hair tip, which consequently creates a softened zone to promote unidirectional expansion [12, 13]. Lateral expansion along the shank of the hair is restricted by the production of a secondary cell wall containing an organized helicoid arrangement of microfibrils that make the wall resistant to turgor pressure [14–17]. To date, only limited information is available concerning the non-cellulosic components that play key roles in tethering microfibrils, and that form the matrix in which the cellulose is embedded [18, 19]. This results in an incomplete understanding of the root hair cell wall composition/organization and its required role in polar expansion.

Over the past two decades, the use of monoclonal antibodies (mAbs) with specificity toward epitopes of various cell wall polymers has greatly enhanced our understanding of cell wall chemistry. Employment of mAbs in high throughput microarrays allows for rapid screening of large numbers of polymers in different taxa, tissues, cell types and cell wall fractions [20, 21]. Similarly, mAbs have been important in mapping specific polymers in various plants and parts therein using light microscopy (LM)-based immunofluorescnce and transmission electron microscopy (TEM)-based immunogold labeling [22–24]. For virtually all of these studies, cells and tissues are typically fixed using chemical or cryofreezing methods, dehydrated and embedded in a plastic or wax matrix that allows for subsequent sectioning prior to imaging [25]. While these protocols have yielded valuable results, they may cause extraction of polymers, artefact formation and wall damage during preparation. More importantly, these techniques only provide snapshots of the cell wall and limited developmental information dealing with the dynamics of the wall in a live plant. Recently, we have devised live-labeling strategies for green algae using mAbs and other molecular probes [26, 27]. These have provided detailed information about wall architecture and wall development during cell expansion in live cells. In this paper, we report on the development of live cell imaging protocol for root hair development in *Arabidopsis thaliana*. This methodology allows for rapid mapping of cell wall polymers using mAbs with live root hairs, and provides a valuable new technique when coupled with transgenic cell lines for detailed studies dealing with wall deposition, secretory dynamics and cell expansion.

## Results

**Figure 1:**
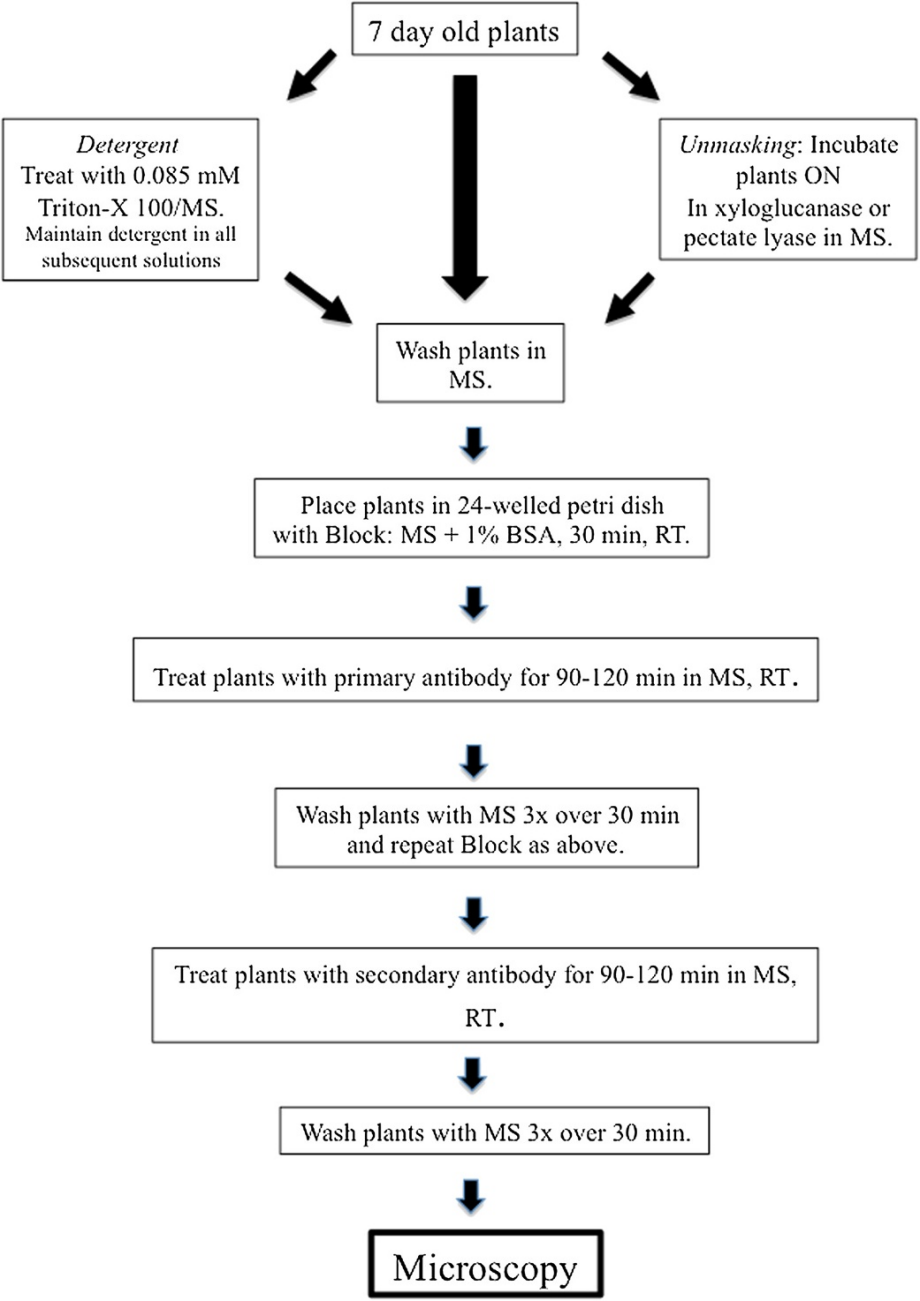
**Schematic outline of live cell labeling protocol.**

The protocol developed here for live labeling of root hairs is both simple and convenient, and is outlined in Figure 1. The elimination of fixation, dehydration and embedding is advantageous especially for handling such fragile specimens as root hairs. This methodology also does not require sectioning or dewaxing protocols and allows for 3-dimensional image acquisition via confocal laser scanning microscopy (CLSM). The use of multi-welled petri dishes allowed for assessment of large numbers of roots in small volumes of labeling solutions of 500 μL or less. Root hairs could also be treated with wall-degrading enzymes to remove and unmask specific polymers prior to labeling [28, 29]. We also tested root hair viability, labeling quality/intensity with and without the inclusion of a detergent (0.85 mM Triton-X100) in the labeling protocol and noted no discernable difference in labeling results. Control experiments also showed that the labeling was specific.

Table 1 provides a summary of antibodies used and labeling results that were obtained for the following phenotypes: wild-type (wt), the *vti13* single mutant, *prp3* single mutant, the *prp3 vti13* double mutant, *csdl2* single mutant, the *csdl3-2* double mutant and the *xxt1*, *xxt2* and *xxt5* single mutants. In wt root hairs, intense labeling was noted for mAbs specific for epitopes of xyloglucan, xylan and arabinogalactan protein with moderate labeling for extensin and homogalacturonan (HG), specifically low esterified HG. Similar results were observed for the *prp3* mutant walls. In *vti13* seedlings, xyloglucan- and xylan-epitope labeling was absent in root hairs. For the double mutant, *prp3 vti13*, xyloglucan and xylan labeling was present in root hairs. The *csld*2-1 mutant produced notably small root hairs [30] while the *csld3-2* mutant produced no root hairs [30]. The *csld2-1* mutant had similar labeling to the wt except for the absence of LM19-probed HG and LM11-probed ß(1–4)-xylan/arabinoxylan. The *xxt* mutants yielded similar results except for the lack of LM10 labeling in the *xxt1* line. Most notable though was the complete lack of labeling with the xyloglucan probes, LM15 and LM25 in all three mutant lines. These results provide evidence that differences in root hair cell wall organization or composition in different genetic backgrounds can be used to identify root hair mutant phenotypes in live seedlings.

**Table 1:** **Summary of antibodies employed and results of immunocytochemical labeling**

mAb	Specificity/Reference	WT	vti13	prp3	prp3/vti13	csld2	csld3-2	xxt1	xxt2	xxt5
***PECTINS***										
JIM7	**High DE HG** [31]	-	-	-	-	-	NA	-	-	-
LM19	**Low DE HG** [23]	++	++	+	++	-	NA	+	+	+
LM5	**(1–4)-galactan** [32]	-	-	-	-	-	NA	-	-	-
LM6	**(1–5)-arabinan** [33]	-	-	-	-	-	NA	-	-	-
INRA-RU1	**Rhamnogalacturonan-I** [34]	-	-	-	-	-	NA	-	-	-
***XYLOGLUCAN***										
LM15	**xylosylated XyG** [29]	++	-	++	++	+	NA	-	-	-
LM25	**galactosylated XyG epitope** [20]	++	-	+	++	+	NA	-	-	-
***MANNAN***										
BS-400-4	**ß(1–4)-mannan/galacto-ß(1–4)-mannan** [35]	-	-	-	-	-	NA	-	-	-
***XYLAN***										
LM10	**(1–4)-xylan** [36]	++	-	++	++	++	NA	-	++	++
LM11	**ß(1–4)-xylan/arabinoxylan** [36]	+	-	+	+	-	NA	-	-	-
***AGP***										
LM2	**AGP** [37]	-	-	-	-	-	NA	-	-	-
JIM13	**AGP** [38]	+	+	+	+	+	NA	+	+	+
JIM8	**AGP** [39]	+	+	+	+	+	NA	+	+	-
***EXTENSIN***										
LM1	**Extensin** [40]	++	++	+	++	+	NA	+	+	+
JIM20	**Extensin** [41]	+	+	+	+	+	NA	+	+	+

Key: ++ = intense labeling, + = moderate labeling, - = ss no label, NA = not applicable, no root hairs formed.

**Figure 2:**
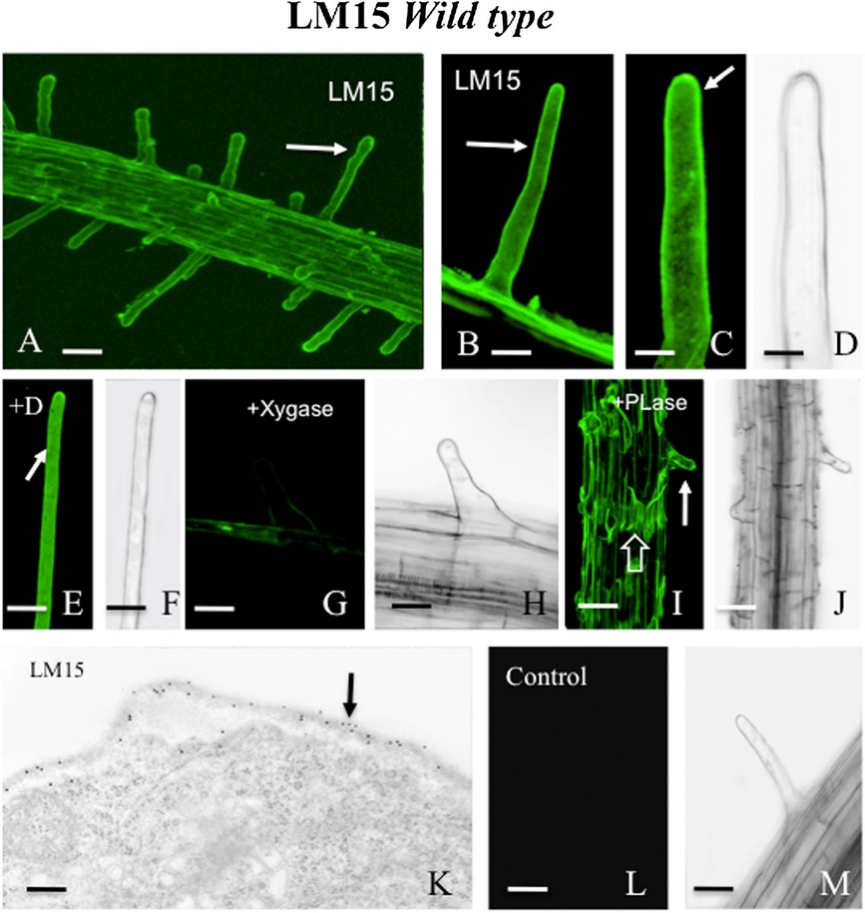
**Live cell LM15 labeling of wild type (wt) Arabidopsis root hairs. A**: Overview of live root demonstrating strong LM15 labeling of root hairs (arrows) and epidermis (hollow arrow). Bar = 100 μm. **B**: Magnified view of a root hair labeled with LM15 (arrow). Bar = 19 μm. **C** and **D**: LM15 labeling of root hair highlighting a slight but notable gradient with more intense labeling at the root hair tip (C, arrow). D is the DIC profile of C. Bars for C and D = 12 μm. **E** and **F**: LM15 labeling of root hair (E, arrow) after incubation of root in 0.085 mM Triton-X detergent (+D), during labeling process. Note labeling of root hair is comparable to that observed with non-detergent labeling (A-C). F is the DIC profile of E. Bars for E and F = 30 μm. **G** and **H**: LM15 labeling after root was pre-incubated overnight in 5U of xyloglucanase (+Xygase). Note the lack of labeling of root and hairs (G). H is the DIC profile of G. Bars for G and H = 18 μm. **I** and **J**: LM15 labeling after root was pre-incubated overnight in 3U of pectate lyase (+PLase). Note that the root hair labels (I, solid arrow). The epidermal cells labeling appears in strips (I, hollow arrow) but still labels. J is the DIC profile of I. Bars for I and J = 50 μm. **K**: Immunogold profile of root hair cell wall labeled with LM15 (arrow), Bar = 50 nm. **L** and **M**: Control profile whereby the primary antibody was eliminated during labeling. M is the DIC profile of L. Bars for L and M = 30 μm.

**Figure 3:**
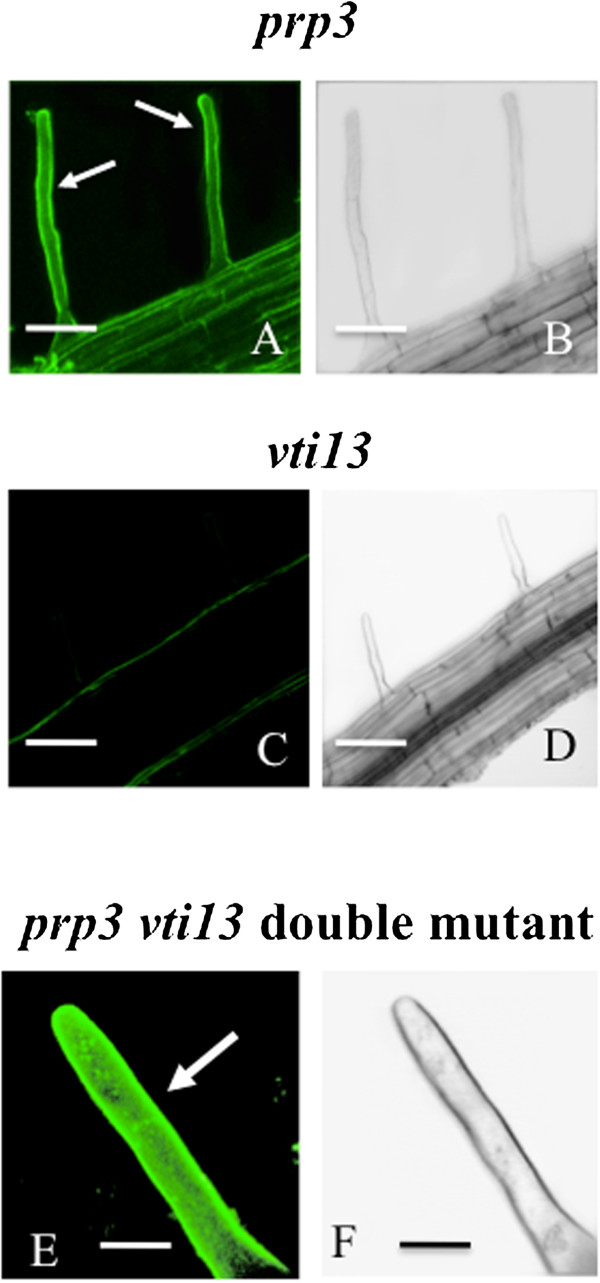
**LM15 labeling of live cells of** ***prp3, vti13,*** **and** ***prp3/vti13*** **double mutant seedlings. A** and **B**: LM15 labels the hairs of the *prp3* mutant. B is the DIC profile of A. Bars for A and B = 50 μm. **C** and **D**: LM15 does not label the root hairs of the *vti13* mutant. D is the DIC profile of C. Bars for C and D = 100 μm. **E** and **F**: In the *prp3/vti13* double mutant, LM15 labels the root hair (E, arrow). F is the DIC profile of E. Bars for E and F = 20 μm.

For detailed analysis of imaging, we chose the xylosylated-xyloglucan specific mAb, LM15 [29]. LM15 labeled the root hairs and epidermis of the wt root (Figure 2A, B) with a subtle gradient of higher labeling at the tip and lesser intensity at the base (Figure 2C). No discernable difference was noted in labeling when the protocol included the detergent-containing base, MS-Tri-X100 (Figure 2E, F). When pre-treated with 5U of xyloglucanase for 90 min, the root hairs did not label with LM15 (Figure 2G, H). When pre-treated with 3U of pectate lyase for 90 min, LM15 labeling was unaltered (Figure 2I, J). TEM immunogold labeling supplemented our CLSM imaging and demonstrated that LM15 labeling was indeed localized at the cell wall (Figure 2K). A control experiment whereby the primary antibody was eliminated from the protocol showed no labeling (Figure 2L, M). LM15 labeled the cell walls of the root hairs of the *prp3* mutant (Figure 3A, B). LM15 did not label the root hairs of the *vti13* mutant (Figure 3C, D) but did label the double mutant, *prp3 vti13* (Figure 3E, F).

**Figure 4:**
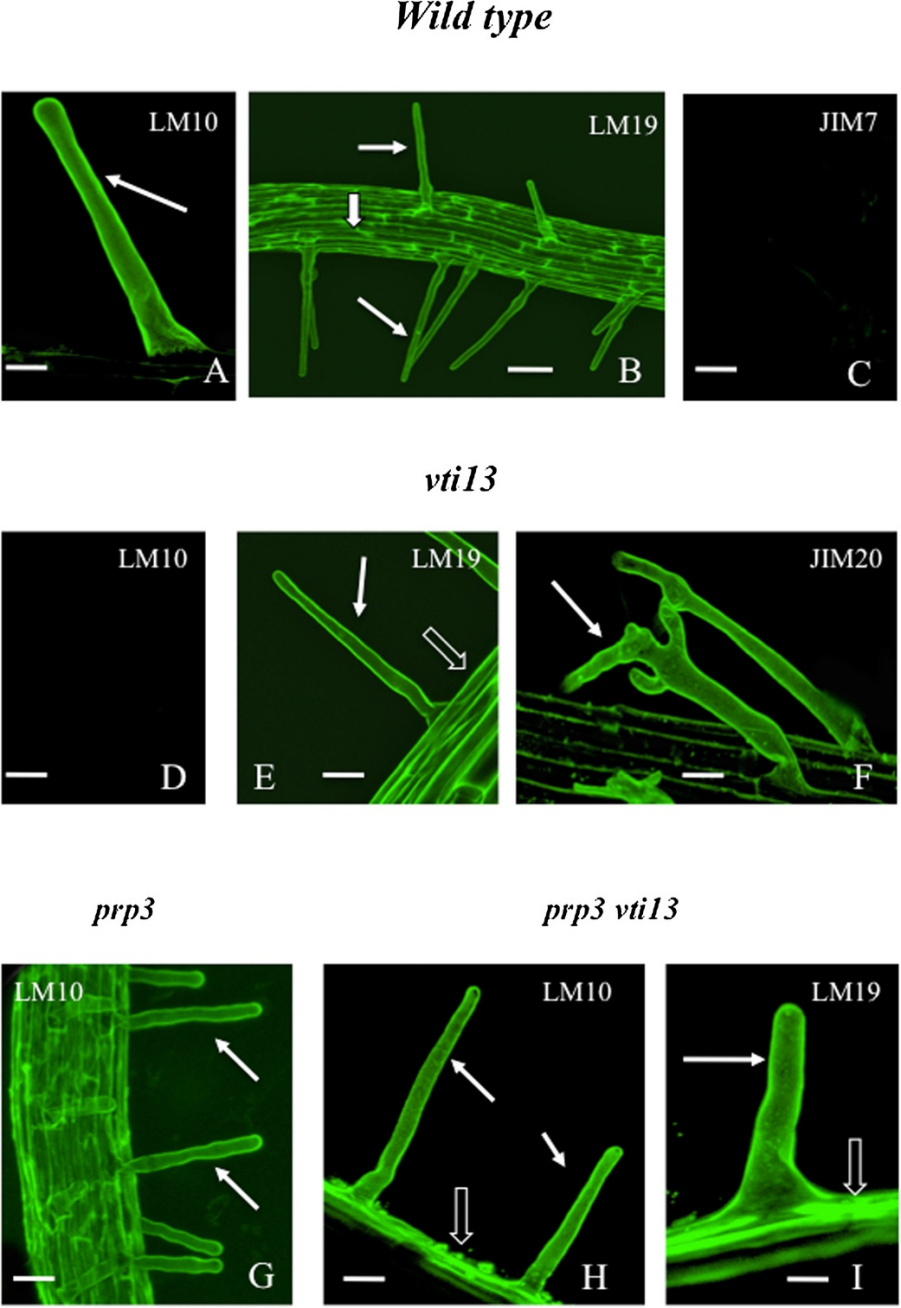
**Live cell profiles of immunolabeled root hairs. A**: LM10 labeling of a wt root hair (arrow). Bar = 10 μm. **B**: LM19 labeling of wt root hairs (solid arrows) and root epidermis (hollow arrow). Bar = 80 μm. **C**: Lack of JIM7 labeling of wt root hairs. Bar = 20 μm. **D**: The root hairs of the *vti13* mutant do not label with LM10. Bar = 30 μm. **E**: LM19 labeling of *vti13* root hairs (solid arrow) and root epidermis (hollow arrow). Bar = 35 μm. **F**: JIM 20 labeling of root hairs of the *vti13* (arrow). Bar = 15 μm. **G**: LM10 labeling of root hairs (arrows) of *prp3* mutant. Bar = 15 μm. **H**: LM10 labeling of root hairs of *prp3/vti13* (solid arrows) and root epidermis (hollow arrow). Bar = 15 μm. **I**: LM19 labeling of root hairs of *prp3/vti13* (solid arrows) and root epidermis (hollow arrow). Bar = 10 μm.

LM10, a mAb with specificity toward xylan, labeled the root hairs of the WT, *prp3* and double mutant, *prp3 vti13,* but not the *vti13* mutant (Figure 4A, D, G, H). Labeling of pectin with relative low levels of esterification using LM19 demonstrated that labeling was present in all genotypes (Figure 4B, E, I; Table 1). Interestingly, JIM7, a mAb with specificity toward high-esterified HG, did not label the root hairs of any of the tested genetic backgrounds (e.g. Figure 4C; Table 1). Previous studies, using fixed sections of plant roots have shown that both JIM5 and JIM 7 label the cell walls of root hairs [42]. These results suggest that the pectin epitopes recognized by JIM5 and JIM7 may not be accessible to the antibody in growing root hairs when using live seedlings. JIM20, a mAb with specificity toward extensin, labeled the root hairs of all genotypes as exemplified by *vti13* (Figure 4F; Table 1). These results help define the dynamics of the root hair cell wall and how organization or composition is altered in root hair mutants, *prp3* and *vti13*.

**Figure 5:**
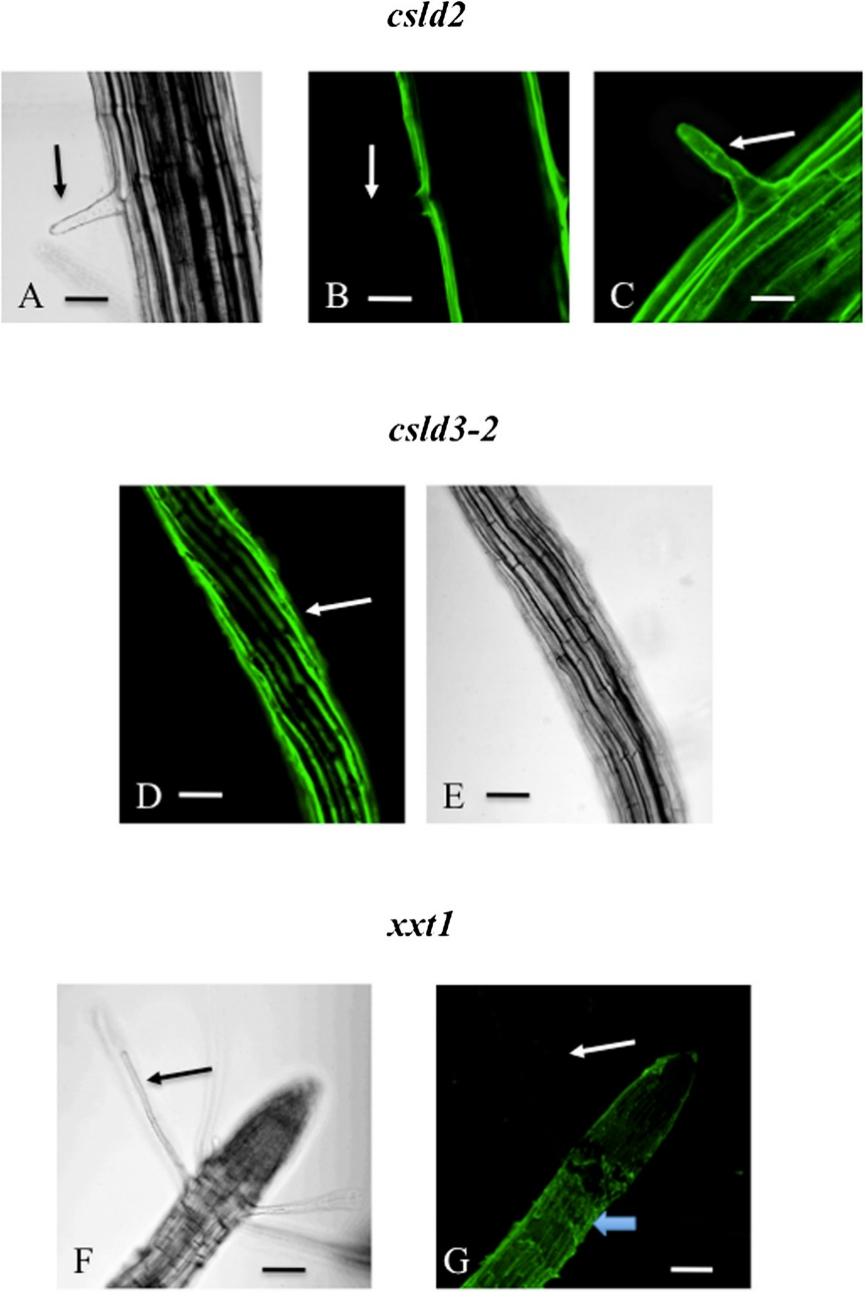
**Live cell profiles of immunolabeled root hairs of cell wall mutant lines. A**: A small root hair of the *csld-2* line located approximately 2 mm from the root tip. Bar = 20 μm. **B**: LM15 labeling of the root hair identified in “A”. Note the absence of labeling (arrow). Bar = 20 μm. **C**: LM15 labeling of a small root hair (arrow) located just behind the root tip of the *csld2* mutant. Bar = 12 μm. **D**: No root hairs were found in the *csld3-2* mutant but the epidermis labeled with LM15 (arrow). Bar = 20 μm. **E**: DIC image of the root shown in “D”. Bar = 20 μm. **F**: The root hairs of the *xxt1* mutant line (arrow). Bar = 30 μm. **G**: LM15 labeling of the root hairs (arrow) observed in “F”. Bar = 15 μm.

These methods were then used to investigate the root hairs of mutant lines defective in the synthesis of structural carbohydrates within the cell wall. The *csld2-1* mutant produced small root hairs that labeled inconsistently with LM15 or LM25. The developing root hairs near the root tip labeled but no label was noted in fully expanded hairs further removed from the root tip (Figure 5A-C). No hairs were formed in the *csld3-2* line as has been previously described [31, 43–45], but it is important to note that LM15/LM25 labeling was noted in the epidermis (Figure 5D, E). For the *xxt* mutant lines, root hairs were present but did not label with either LM15 or 25, as illustrated for *xxt1* (Figure 5F, G). In summary, the results of this study highlight a convenient and simple means to identify other cell wall mutant lines and better define changes in their wall microarchitecture.

## Discussion

Live cell labeling of root hairs of Arabidopsis provides a valuable new tool in elucidating wall microarchitecture and development. This simple and relatively fast method of mapping specific wall polymers offers much potential in adding detail to cell wall deposition/organization and cell expansion dynamics. In this study, we demonstrated labeling of xyloglucans in the root hairs of WT, *prp3* single mutant and the double mutant, *prp3 vti13* using the xylosylated-xyloglucan specific mAb, LM15, and the galactosylated xyloglucan specific mAb, LM25. These observations supplement recent research where a galacturonic acid-containing xyloglucan is found in *Arabidopsis* root hair cell walls [18]. Likewise, we show a higher intensity labeling at the root tip in our study with LM15 in WT and *prp3 vti13* double mutant root hairs compared to the labeling in the *vti13* single mutant, which has a branching root hair phenotype under our growth conditions [46]. This observation suggests that xyloglucan polymers may play a key role in providing controlled tensile resistance to turgor pressure at the root hair tip during growth. This tensile support might be especially important in this zone where less organized cellulose microfibril organization has been noted [12]. Our study has also shown that wall glycoproteins such as extensin and arabinogalactan proteins can be detected in the root hair wall of live seedlings, supporting recent work that also showed the presence of these polymers in the wall [19, 47, 48].

In the *Arabidopsis* root hair, xyloglucan and other hemicellulosic polymers like xylan may also be critical to regulating wall extensibility. The absence of labeling of certain epitopes in the *vti13* single mutant and the recovery of these labeling patterns in the *prp3 vti13* double mutant suggests that there is a reorganization of the cell wall matrix in *vti13* root hairs, rather than a loss of these components from the cell wall. Therefore, the *vti13* mutant has an altered deposition or development of the cell wall such that the epitopes are not available to the mAb labeling. The reemergence of the epitopes in the *prp3 vti13* is evidence that the cell wall has gone through another reorganization, not a loss of specific constituents of the matrix.

Our results also show that this methodology can also be used for studying wall mutant lines, especially those deficient in the synthesis of specific wall polymers. The *xxt* mutants did not label with LM15 or LM25, antibodies specific for xyloglucan epitopes. Genetic analysis of *xxt1* and *xxt2* mutants has shown that xyloglucan synthesis is required for root hair growth [42, 49]. In addition, similar amounts of xyloglucan can be detected within the walls of wt and single *xxt1* and *xxt2* mutant seedlings using OLIMP analysis, suggesting that these two loci compensate for one other during xyloglucan synthesis [42]. The lack of labeling of root hairs in *xxt* mutant seedlings with LM15/LM25 in this study suggests subtle changes in xyloglucan orientation within the wall may eliminate the availability of the LM15 and LM25 epitopes using live seedlings. For the *csld* mutants, lines deficient in the synthesis of ß-glucans (e.g. mannose, cellulose; [30, 50, 51]), the results were limited as the two lines either did not produce root hairs or yielded small hairs with inconsistent labeling. Overall, the ability to discern these nuances of cell wall metabolism makes this live labeling protocol a powerful tool in defining phenotypes in cell wall and root hair mutants. A next step in expanding use of this method will be labeling with two or more wall-specific mAbs.

## Conclusion

Live cell labeling of cell wall epitopes allows for dynamic imaging of wall structure during development as well as important application for co-labeling studies. For example, using this methodology along with fluorescent protein-fusion constructs of various components of the secretory and cytoskeletal machinery of the hair offer powerful and specific mechanisms for deciphering the coordinated interactions of specific subcellular systems in the development of the cell wall and constituent polymers therein.

## Methods

### General

Seeds of wt and various Arabidopsi*s* mutants (all in a Columbia background) were surface sterilized with a 20% (v/v) bleach solution and sown on MS medium (1X Murashige and Skoog salts, 1% (w/v) sucrose, 1X Gamborg’s vitamin solution, 5 mM MES pH6) and solidified with 1.3% (w/v) agarose as described in Larson et al. [36]. The *vti13* mutant (SALK_075261) was obtained from the Arabidopsis Biological Research Center (ABRC) and confirmed to be a null mutant. The *prp3* and *vti13* single mutants were crossed using standard procedures and the homozygous double mutant was identified in the F2 generation using genomic PCR (described in [36]). CSLD- and XXT-mutant lines were a generous gift of Dr. Ken Keegstra. Murashige-Skoog (MS) medium was obtained from Sigma Chemical (St. Louis, MO, USA) and antibodies were obtained from Plant Probes (Leeds, UK), Sigma Chemical (St. Louis, MO, USA) and a generous gift from Dr. Marie Christine-Ralet (INRA, Nantes, FR).

### Immunofluorescence labeling

All labeling was performed in a 24-welled petri dish (non-tissue culture treated dishes; Fisher Scientific, Pittsburg, PA, USA). Arabidopsis seedlings were grown vertically on MS-containing media for 7 days after which they were gently removed from the agarose surface, placed in the wells of the petri dish and incubated for 30 min in 500 μL of liquid MS medium containing 1.0% (w/v) non-fat Carnation Instant milk. This represented the blocking solution for immunolabeling. In an initial test of labeling quality and root hair viability, we compared samples processed in MS as the buffer for labeling with samples processed in MS and detergent (0.85 mM Triton-X100). No difference was noted between the two solutions and the inclusion of detergent was maintained for all subsequent labeling and is noted as MS-Tri-X100 below. After blocking, the seedlings were washed 3X over 30 min with liquid MS medium and then placed in 500 μL of primary antibody solution that consisted of a 1/10 (v/v) dilution of wall polymer-specific antibody in MS-Tri-X100. Plates were gently shaken on a laboratory rotator for 90 min in the dark at Room Temperature (RT). Seedlings were again washed 3X with MS-Tri-X100, blocked (as described above) and washed again. Seedlings were incubated and gently shaken for 90 min in the dark at RT in 500 μL of a 1/75 (v/v) dilution of secondary antibody (anti-rat-TRITC; Sigma) in MS-Tri-X100. They were then washed with liquid MS 3X over 30 min. The seedlings were left in MS liquid medium until microscopic viewing. For control, primary antibody labeling was eliminated from the protocol. For each primary antibody tested at least 10–15 root hairs of 3 separate seedlings were observed. This process was also performed three times.

For unmasking experiments, some seedlings were gently shaken in solutions of 5U xyloglucanase (Megazyme, IR) or 3U pectolyase (Megazyme) in MS for 90 min at RT. The seedlings were then removed, washed 3X with MS and processed for labeling (see above).

The labeled seedlings were placed in a 200 μL drop of liquid MS in the well of a single-welled immunoslide (EMS, Ft. Washington, PA, USA). A glass coverslip was placed gently over the seedling. The depression of the immunoslide prevented crushing of the root and hairs. For some slides, the coverslip was affixed to the slide with small drops of nail polish. Root hairs were then observed using an Olympus BX-61 microscope equipped with a Fluoview 300 confocal system. The fluorescence signals were also observable using wide field Olympus BX-60 or IX70 fluorescence microscopes with 50 watt mercury lamp[s and TRITC filter sets.

### Transmission Electron Microscopy (TEM)

Roots were fixed in 0.5% (v/v) glutaraldehyde (EMS, Ft. Washington, PA, USA) in Sorensen’s Phosphate buffer (EMS; pH 7.2) for 40 min at 4°C. They were washed 3X with Sorensen’s buffer and post-fixed in 0.5% (w/v) OsO_4_ (EMS) in Sorensen’s buffer for 1 h at 4°C. After washing with buffer (see above), the seedlings were dehydrated in acetone and embedded in Spurrs Low Viscosity plastic. 60–80 nm sections were obtained using a Reichert Ultracut ultramicrotome and collected on formvar coated nickel grids. Immunogold labeling was performed using previously developed techniques [52].
